# Relationship between changes in microbiota induced by resveratrol and its anti-diabetic effect on type 2 diabetes

**DOI:** 10.3389/fnut.2022.1084702

**Published:** 2023-01-06

**Authors:** Alfredo Fernandez-Quintela, María Teresa Macarulla, Saioa Gómez-Zorita, Marcela González, Iñaki Milton-Laskibar, María P. Portillo

**Affiliations:** ^1^Nutrition and Obesity Group, Department of Nutrition and Food Science, University of the Basque Country (UPV/EHU) and Lucio Lascaray Research Institute, Vitoria-Gasteiz, Spain; ^2^Bioaraba Health Research Institute, Vitoria-Gasteiz, Spain; ^3^CIBERobn Physiopathology of Obesity and Nutrition, Institute of Health Carlos III, Vitoria-Gasteiz, Spain; ^4^Nutrition and Food Science Department, Faculty of Biochemistry and Biological Sciences, National University of Litoral and National Scientific and Technical Research Council (CONICET), Santa Fe, Argentina

**Keywords:** resveratrol, type 2 diabetes, insulin resistance, anti-diabetic effect, gut microbiota

## Abstract

Although a general healthy gut microbiota cannot be defined due to numerous internal and external individual factors, such as sex, age, ethnicity, genetics, environment, diet and drugs affect its composition, certain microbial species and gut microbiota compositions seem to be related to the progression of insulin resistance to type 2 diabetes, as well as the development of microvascular and macrovascular complications of diabetes. The present review aimed at gathering the reported information describing how resveratrol induced changes in microbiota composition can mediate the positive effects of this polyphenol on glucose homeostasis under type 2 diabetic conditions, both in animals and humans. Based on the fact that some changes observed in the gut microbiota of type 2 diabetic animals and patients are reversed by resveratrol treatment, and taking into account that some resveratrol mediated changes in gut microbiota composition are similar to those induced by anti-diabetic drugs such as metformin, it can be proposed that four genera, *Alistipes, Allobaculum, Desulfovibrio* and *Blautia* could be involved in the benefits of resveratrol on glycameic control. Nevertheless some limitations are observed in this research field: (a) the number of studies analyzing both the effects of resveratrol on glucose homeostasis and microbiota composition in the same cohort of animals, in order to know the potential involvement of microbiota in the anti-diabetic effects of this phenolic compound, are very scarce and practically inexistent in the case of humans., (b) the studies present inconsistencies concerning the effects of resveratrol on gut microbiota changes, (c) the experimental design used do not allow the researchers to establish a causal relationship between the changes in microbiota and the anti-diabetic effect, in the vast majority of the studies, (d) the knowledge about the role of each type of bacteria on glycaemic control is not sufficient so far.

## Introduction

Type 2 diabetes mellitus is a non-communicable disease characterized by an elevated blood glucose level or hyperglycaemia. Several risk factors such as a family history of diabetes, poor eating habits and being overweight or obese have been identified. In 2019, about 463 million people suffered diabetes worlwide, and future estimates predict that by 2045, the number of diabetic patients will exceed 700 million (1).

The intestinal microbiota could be defined as the complex community of microorganisms that inhabit our gastrointestinal tract (2, 3). It is mainly composed of bacteria, with the most common being the phyla *Bacteroidetes*, *Clostridium*, *Prevotella*, *Eubacterium*, *Ruminococcus*, *Fusobacterium*, *Peptococcus* and *Bifidobacterium* (4). In addition, other microorganisms, such as viruses and fungi are also part of the intestinal microbiota, although in a much smaller proportion. It is known that gut microbiota plays a major role in various biological processes as well as being involved in the development of several diseases (5). Indeed, although a general healthy gut microbiota cannot be defined due to the fact that numerous internal and external individual factors, including sex, age, ethnicity, genetics, environment, diet and drugs affect its composition (6–9), certain species and combinations of them seem to be related to, or associated with specific diseases (10).

Regarding type 2 diabetes mellitus and insulin resistance, some studies have revealed a significant influence of gut microbiota on insulin signaling, inflammation, and glucose homeostasis (11–13). In addition, it has been reported that dysbiosis (the impairment of gut microbiota composition and gut barrier function) accompanies the progression of insulin resistance to type 2 diabetes, as well as the development of microvascular (retinopathy, nephropathy, and neuropathy) and macrovascular (atherosclerosis) complications of diabetes (14). The dysbiosis found in type 2 diabetic subjects is characterized by a decrease in butyrate-producing bacteria and an increase in opportunistic pathogens (11). Dysbiosis also involves the modification of intestinal integrity, islet inflammation and insulin signaling, at least in part, by means of metabolites produced by bacteria (short-chain fatty acids or secondary bile acids), which interact with receptors on epithelial, fat, muscle, liver, pancreatic and cardiac cells.

Very recently, Letchumanan et al. (13) have published a systematic review devoted to analyzing the existing evidence related to gut microbiota composition and diversity in individuals with pre-diabetes and newly diagnosed individuals with type 2 diabetes mellitus, in comparison to control subject. In this review, the authors pointed out that, despite the studies found significant associations between clinical biomarkers and the abundance of specific bacterial groups, a great heterogeneity in methodology and inconsistencies in the results were found among the eighteen studies gathered, with the exception of the correlation between glycaemic markers and *Lactobacillus* abundance. By focusing on changes that were similarly reported in two or more studies, they observed that the abundance of certain bacteria was increased (*Lactobacillus, Streptococcus*, *Escherichi*a, *Veillonella* and *Collinsella*) or decreased (*Roseburia*, *Dialister*, *Flavonifractor, Alistipes, Haemophilus* and *Akkermansia muciniphila*) in prediabetic and diabetic subjects. In other studies, high relative abundances of some opportunistic pathogens, such as *Bacteroides caccae, Clostridium hathewayi, Clostridium ramosum, Clostridium symbiosum*, and *Escherichia coli*, as well as low relative abundances of butyrate-producing bacteria (*Roseburia intestinalis* and *Faecalibacterium prausnitzii*) have been observed (15, 16). Moreover, some studies carried out in rodents have confirmed that the abundances of *Allobaculum* and *Lactobacillus* diminish whereas those of *Bacteroides*, *Veillonella*, *Lachnoclostridium*, *Parasutterella*, *Bifidobacterium*, *Helicobacter*, *Cupriavidus*, *Halomonas*, *Odoribacter*, *Enterococcus* and *Shigella* increase in type 2 diabetic animals (17–19).

As previously indicated in this section, microbiota composition very much depends on the diet. In particular, polyphenols, which are chemical compounds naturally present in some foodstuffs, such as fruits, vegetables and nuts, among others, have been demonstrated to strongly interact with microbiota, thus leading to significant changes on its composition. In addition, these compounds, among which resveratrol (3,5,4’-trihydroxy-trans-stilbene) stands out, have shown beneficial effects on health in numerous pre-clinical studies, as well as in epidemiological studies and clinical trials.

**FIGURE 1:**
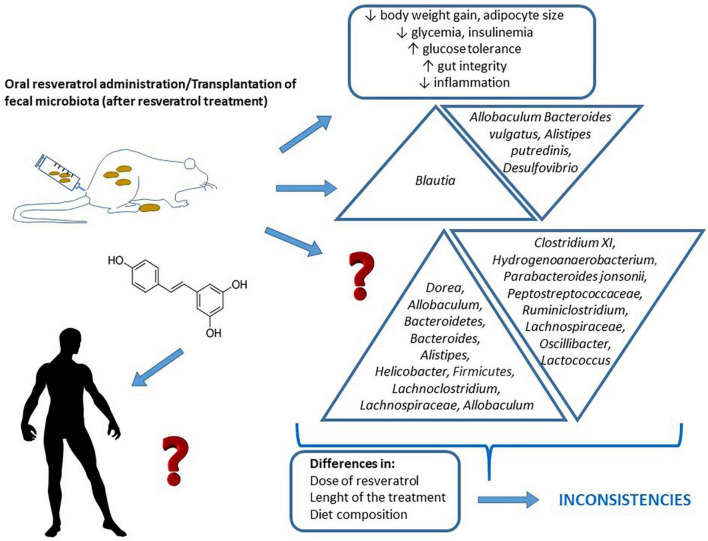
Changes induced by resveratrol administration, or fecal matter transplantations from animals treated with resveratrol, on glycaemic control and gut microbiota composition. The figure also shows the differences among experimental designs that sometimes lead to insistencies in the obtained results, and the lack of studies addressed in humans.

Regarding type 2 diabetes mellitus, it has been reported that resveratrol improves insulin sensitivity and reduces blood glucose in cell cultures and animal models (20). The recent meta-analysis published by García-Martínez et al. (21) shows that in several clinical trials, resveratrol increases insulin sensitivity and decreases blood glucose levels either in subjects featuring insulin resistance or type 2 diabetes mellitus (22, 23; Figure 1). However, a general consensus has not been achieved since there are also some clinical trials in which these positive effects of resveratrol have not been observed. These discrepancies have been attributed to the wide range of doses used (5–5,000 mg/day), as well as to other factors, such as the age and the metabolic status of the participants. The general conclusion of this meta-analysis is that resveratrol has a significant dose-response effect on glucose concentrations, HbA1c percentage and insulin levels in subjects with type 2 diabetes mellitus, aged 45–59 years.

Among the mechanisms underlying the anti-diabetic effects of resveratrol, increased insulin sensitivity, activation of 5′-AMP-activated protein kinase (AMPK), epigenetic modifications of DNA sequence (including methylation and histone modifications) leading to changes in gene expression, and improvement of pancreatic β-cell functionality, due to the protection against oxidative damage, have been described (24–27). In addition, several authors have proposed that changes induced by resveratrol in gut microbiota composition may well be involved on its anti-diabetic effect.

In this context, the aim of the present review is to gather the information reported so far describing how the changes induced by resveratrol in gut microbiota composition can mediate the positive effects of this polyphenol on glucose homeostasis under type 2 diabetic conditions, both in animals and humans.

## Animal studies addressing the effects of resveratrol administration on glycemic control and gut microbiota

Although there are a great number of studies aimed at analyzing the effect of resveratrol on glycaemic control or to determine how resveratrol administration modifies gut microbiota, only a reduced number of such studies have analyzed both aspects at the same time (in the same cohort of animals), in order to shed light onto the involvement of microbiota changes in the anti-diabetic effect of this polyphenol. These studies have been carried out in both mice and rats (Table 1 and Figure 1).

**TABLE 1:** Effects of resveratrol administration on glycaemic control and gut microbiota composition.

References	Experimental design	Main results concerning glycaemic control	Main results concerning microbiota
Dao et al. (28)	Animals: C57Bl/6J wild mice and Glp1r^–/–^ mice Age and sex: 8-week-old; male Diets: standard diet (12 kcal% fat) or high-fat diet (72 kcal% fat) Experimental period length: 5 weeks Dose of resveratrol: 60 mg/kg BW/day	Wild type mice: ↓ Reduced glucose intolerance Reversion of the reduction in GLP-1 receptor levels Glp1r^–/–^ mice: No effects	Disappearance of the species: *Parabacteroides jonsonii* *Alistipes putredinis* *Bacteroides vulgatus*
Etxeberria et al. (34)	Animals: Wistar rats Age and sex: 6-week-old; male Diets: high-fat diet (45 kcal% fat, 35 kcal% CH) Experimental period length: 6 weeks Doses of resveratrol: 15 or 30 mg/kg BW/d	↓ Reduced serum insulin levels ↓ HOMA-IR index	No effects
Jung et al. (29)	Animals: C57BL/6J mice Age and sex: 5-week-old; male Diets: standard diet (12.41 kcal% fat) or high-fat diet (60 kcal% fat) Experimental period length: 8 weeks Dose of resveratrol: 200 mg/kg BW/d	Improvement of glucose tolerance and insulin resistance	↓ *Lactococcus* ↓ *Clostridium XI* ↓ *Oscillibacter* ↓ *Hydrogenoanaerobacterium*
Sung et al. (30)	Animals: C57BL/6J mice Age and sex: 8-week-old; male Diets: 45 kcal% fat, 17 kcal% sucrose Experimental period length: 8 weeks Dose of resveratrol: 0,4% in the diet	*↑ Glucose clearance*	↓ *Turicibacteraceae* ↓ *Moryella* ↓ *Lachnospiraceae* ↓ *Akkermansia* *↑ Bacteroides* *↑ Parabacteroides*
Yang et al. (35)	Animals: Wistar rats Age and sex: 6-week-old; male Diets: a high-fat diet (45 kcal% fat) Experimental period length: 8 weeks Dose of resveratrol: 10 mg/kg BW/d	↓ Fasting blood glucose ↓ Insulin ↓ HOMA-IR	Reversion of the changes induced by high-fat feeding: Disappearance of: *Parabacteroides jonsonii* *Alistipes putredinis* *Bacteroides vulgatus*
Wang et al. (31)	Animals: C57BL/6J mice Age and sex: 6-week-old; male Diets: standard diet (10 kcal% fat) or high-fat diet (60 kcal% fat) Experimental period length: 16 weeks Dose of resveratrol: 300 mg/kg BW/d	Restoration of fasting blood glucose and insulin concentrations Improvement in insulin resistance and glucose tolerance ↑ SCFA-producing bacteria	↑ *Bacteroidetes* ↑ *Allobaculum* ↑ *Blautia* ↓ *Desulfovibrio* ↓ *Lachnospiraceae* ↓ *Alistipes* ↓ *Ruminiclostridium*
Chen et al. (32)	Animals: C57BL/6J mice Age and sex: 7-week-old; male Diets: standard diet (10 kcal% fat) or high-fat diet (60 kcal% fat) Experimental period length: 12 weeks Dose of resveratrol: 100 mg/kg BW/d	↓ Insulin resistance	↑ *Verrucomicrobia* ↑ *Akkermansia* ↑ *Roseburia* ↑ *Anaerostipes*
Du et al. (33)	Animals: C57BL/6J mice Age and sex: 8-week-old; male Diets: standard diet (10 kcal% fat) or high-fat diet (60 kcal% fat) Experimental period length: 4 weeks Dose of resveratrol: 100 mg/kg BW/d	↓ Fasting blood glucose level ↓ Insulin level ↓ HOMA-IR	↑ *Firmicutes* *↑ Allobaculum* *↑ Actinobacteria* ↓ *Porphyromonadaceae* ↑ *Erysipelotrichaceae* ↓ *Ruminococcaceae* ↑ *Allobaculum* ↑ *Enterorhabdus* ↓ *Intestinimonas* ↓ *Clostridium IV* ↓ *Anaerotruncus* ↓ *Flavonifractor* ↓ *Clostridium XlVb*

BW, body weight; CH, carbohydrates; GLP-1, glucagon-like peptide 1; HOMA-IR, Homeostatic Model Assessment Insulin Resistance; SCFA, short-chain fatty acid.

As far as the studies conducted in mice, Dao et al. (28) studied the involvement of glucagon-like peptide-1 (GLP-1) and its receptor on the effect of resveratrol on glycaemia. For this purpose, they worked with two mice models, wild-type C57Bl/6J and Glp1r-/- (mice with a functional disruption of the Glp1 receptor). One group of each model was fed a standard diet (12% of energy from fat) and another a high-fat diet (72% of energy from fat), which induced glucose intolerance before the onset of obesity, and reduced plasma GLP-1 levels. Half of the animals fed the high-fat diet were treated with resveratrol (60 mg/kg body weight/d), while the other half did not receive the polyphenol. In the wild-type model fed the high-fat diet, the authors observed that resveratrol treatment reduced glucose intolerance (as determined by a glucose tolerance test), tripled the concentration of active GLP-1 in the portal vein, increased intestinal glucagon and active GLP-1 and increased insulin concentration during the glucose tolerance test. In contrast, Glp1r-/- mice were insensitive to resveratrol treatment, revealing an essential role of GLP-1 receptor in the control of glucose tolerance by this polyphenol. In addition, in the peripheral organs, resveratrol reduced inflammation, in part by increasing interleukin 10 (IL-10) production in the colon, liver and muscle.

In terms of gut microbiota, the high-fat diet induced great alterations on its composition, while resveratrol treatment normalized them. In particular, the species *Parabacteroides jonsonii* DMS 18315, *Alistipes putredinis* DMS 17216 and *Bacteroides vulgatus* ATCC 8482, which were present in the animals fed the high-fat diet, disappeared in the resveratrol-treated animals. The authors concluded that the GLP-1 receptor is required to mediate resveratrol anti-diabetic effect, and that the mechanism(s) by which GLP-1 secretion is restored may be related to a change in gut microbiota and inflammation.

In another study, Jung et al. (29) fed C57BL/6J mice either a standard (12.41% of energy from fat) or a high-fat diet (60% of energy from fat) and treated or not with resveratrol (200 mg/kg body weight/d), for 8 weeks. The authors studied the involvement of mammalian target of rapamycin (mTOR), which acts as a tyrosine protein kinase that promotes the activation of insulin receptors and insulin-like growth factor 1 receptors. The results showed that the polyphenol prevented glucose intolerance in mice fed the high-fat diet. Regarding gut microbiota, they observed that the high-fat diet increased the relative abundances of the genera *Lactococcus*, *Oscillibacter*, *Hydrogenoanaerobacterium* and *Clostridium cluster* XI. After resveratrol treatment, the abundance of these bacteria decreased. The authors observed that changes in gut microbiota composition caused by resveratrol treatment correlated with alterations in insulin resistance and gut inflammation, concluding that mTOR signaling appeared to be a key component of the regulation of gut microbiota composition.

In the study reported by Sung et al. (30), C57BL/6N mice were divided into four groups: two groups were fed a standard diet, supplemented or not with resveratrol (0.4% in the diet), and two groups were fed a diet rich in fat and sucrose (HFHS, 45% of energy from fat and 17% of energy from sucrose) supplemented or not with resveratrol (0.4% in the diet). The experimental period was of 8 weeks. Mice fed the HFHS diet showed lower glucose clearance in the glucose tolerance test than animals fed the standard diet, while mice fed the HSHS diet and treated with resveratrol showed an improved glucose clearance (represented by the AUC in glucose tolerance test) than non treated HFHS fed mice. With regard to microbiota composition, the authors observed that resveratrol administration produced several changes: a decreased abundance of the families *Turicibacteraceae*, *Lachnospiraceae* and the genera *Moryella* and *Akkermansia*, and an increased abundance of the genera *Bacteroides* and *Parabacteroides*.

Also using C57BL/6 J male mice Wang et al. (31) carried out an experiment feeding the animals either a standard diet (SD group) or a high-fat diet (HFD group; 60% of energy from fat), and administering (by gavage) resveratrol at a dose of 300 mg/kg body weight/d to the treated groups (groups SDR and HFDR), for 16 weeks. The authors observed that resveratrol supplementation improved insulin resistance in high-fat diet fed mice by restoring fasting blood glucose and insulin concentrations to nearly normal levels. Further, and as expected, microbiota composition also changed after the polyphenol administration. Relative abundances of the phylum *Bacteroidetes* and the genus *Bacteroides* were increased in both resveratrol-treated groups (SDR and HFDR groups). Moreover, mice treated with resveratrol showed lower abundances of *Allobaculum*, *Desulfovibrio*, *Lachnospiraceae_NK4A136*_group and *Ruminiclostridium_9* and higher abundances of *Helicobacter*, *Blautia* and *Lachnoclostridium*.

More recently, Chen et al. (32) determined the effects of resveratrol on gut microbiota composition and intestinal tight-junction proteins in 6-week-old male C57BL/6J mice fed a standard or a high-fat diet (60% of energy from fat). Half of the animals received resveratrol at a dose of 100 mg/kg body weight/day for 12 weeks. Consumption of the high-fat diet induced hyperglycemia and insulin resistance, which were prevented by resveratrol administration. Regarding inflammation, resveratrol improved endotoxemia and secondary systemic inflammation by decreasing bacterial lipopolysaccharide, tumor necrosis factor α (TNF-α) and interleukin 1b (IL-1b). On the other hand, in relation to the structure of the tight junctions, the authors observed a decrease in the expression of the tight junction proteins claudin-1, occludin and Zo-1 in the colon of mice fed high-fat diet, which may contribute to the increased passage of bacterial endotoxins into the circulation. Concerning gut microbiota composition, at the phylum level, feces from the group fed the high-fat diet and treated with resveratrol showed increased abundance of *Verrucomicrobia* and at the genus level increased abundance of *Akkermansia*, *Roseburia* and *Anaerostipes*.

Du et al. (33), carried out a study to test the effects of resveratrol in a non-alcoholic fatty liver disease (NAFLD) mouse model. For this purpose, they used male C57BL/6J mice randomly assigned to the control group (NC, fed a normal chow diet), to the HFD group (fed a high-fat diet providing 60% of energy from fat) or to the RSV group (fed the same high-fat diet and treated with 100 mg/kg body weight/d of resveratrol), for 4 weeks. Resveratrol improved glucose homeostasis by reducing insulin levels, fasting blood glucose, and HOMA-IR. Regarding gut microbiota composition, several changes were induced by the high-fat feeding. Thus, at the phylum level, the relative abundance of *Firmicutes* was significantly increased and that of *Bacteroidetes* significantly decreased. At the family level, *Porphyromonadaceae* was decreased and *Lachnospiraceae* and *Ruminococcaceae* were elevated. At the genus level, decreases in *Barnesiella* and *Parasutterella*, and increases in *Intestinimonas*, *Enterorhabdus*, *Oscillibacter*, *Clostridium IV*, *Pseudoflavonifractor*, *Anaerotruncus*, *Clostridium XlVb*, and *Peptococcus* relative abundances were observed. When comparing gut microbiota composition between HFD and RSV groups, it was observed that resveratrol increased *Firmicutes* abundance at the phylum level and *Allobaculum* abundance at the genus level. Correlation analysis revealed that *Firmicutes*, *Lachnospiraceae*, *Pseudoflavonifractor*, *Intestinimonas*, Ruminococcaceae, *Flavonifractor*, *Clostridium XlVb*, *Peptococcus*, *Oscillibacter*, *Anaerotruncus*, and *Hydrogenoanaerobacterium* were positively correlated to fasting blood glucose and HOMA-IR index, while *Barnesiella*, *Bacteroidetes*, *Porphyromonadaceae*, *Parasutterella*, and *Allobaculum* were negatively correlated.

In addition, other studies have also been addressed in rats. In our laboratory a study was carried out to analyze the effect of two resveratrol doses (15 or 30 mg/kg body weight/d) on glycaemic control in rats fed a high-fat high-sucrose diet (45% of energy as fat, 17 g sucrose/100 g diet) for 6 weeks. Resveratrol administration effectively reduced serum insulin levels and improved insulin resistance (determined by the HOMA-IR index), but no changes in microbiota composition were observed (34).

In the study reported by Yang et al. (35) using rats, the animals were randomly distributed in three experimental groups: a control group fed a standard diet (10% of energy from fat); a group fed a high-fat diet (HF; 45% of energy from fat) and a group, fed a high-fat diet and supplemented with resveratrol (HFR; 10 mg/kg body weight/day). The experimental period length was of 8 weeks. Plasma glucose and insulin levels were reduced after resveratrol supplementation as compared to the non-supplemented group, meaning that insulin resistance was reduced.

Regarding gut microbiota composition, alpha diversity indexes were significantly increased in resveratrol-treated rats. Due to the decline of *Bacteroides*, the relative ratio of *Firmicutes* to *Bacteroidetes* showed an increase in the group treated with resveratrol, when compared to the control and HF groups. At the family level, resveratrol-supplemented rats (HFR group) showed the lowest *S24-7* and *Peptostreptococcaceae* abundances. No significant changes in the relative abundance of *Ruminococcaceae*, *Clostridiaceae*, *Bacteroidaceae* or *Desulfovibrionaceae* were detected among the experimental groups. At the genus level, the HFR group was enriched in the butyrate producer *Blautia* and *Dorea* when compared to the HF group, which contributed to a sharp increase in the *Lachnospiraceae* family.

## Human studies addressing the effects of resveratrol administration on glycemic control and gut microbiota

Walker et al. (36) investigated whether the administration of high doses of resveratrol to obese individuals featuring insulin resistance and metabolic syndrome could improve certain parameters such as insulin sensitivity and glucose tolerance. Moreover, they also analyzed intestinal microbiota composition. For this purpose, obese men with a body mass index of 30–40 kg/m^2^ and aged between 30 and 70 years were studied. The work was conducted in two experimental groups using a placebo-controlled, double blind, randomized, parallel-group pilot study design. The patients were fed for 4 days an isocaloric Western diet, providing the calories needed to maintain a stable weight. On day four and for 30 days, twice daily, subjects in the resveratrol group began receiving two 500 mg capsules of Mega-RES 99% (made exclusively from organically grown Japanese root) and those in the placebo group, two 500 mg placebo capsules. Only minor overall effects of resveratrol were demonstrated in obese men with insulin resistance and metabolic syndrome. Resveratrol did not induce changes in insulin resistance, but it did reduce the 120-min time point and the area under the 2-h glucose tolerance test glucose concentration curve. Several changes in gut microbiota composition were also induced by the polyphenol. Thus, a drop in the relative abundances of family *Rikenellaceae* and the genera *Ruminococcus*, *Oscillospira*, *Clostridium*, *Alistipes*, *Odoribacter* and *Butyricimonas* were found. Moreover, the relative abundances of the phylum *Gammaproteobacteria*, the family *Gemellaceae* and the genera *Turicibacter* and *Atopobium* were increased.

## Animal studies addressing the effects of fecal transplantation from resveratrol-treated animals on glycemic control and gut microbiota

The studies described in the previous section do not establish a causal relationship between changes in microbiota and changes in glucose homeostasis induced by resveratrol. However, in the literature, there are some studies in which fecal matter transplantations from animals treated with resveratrol to non- treated animals, showing alterations in glucose homeostasis, have been carried out to gain more insight about the potential involvement of microbiota in the anti-diabetic effects of resveratrol (Table 2 and Figure 1).

**TABLE 2:** Effects induced by fecal matter transplantations from animals treated with resveratrol on gut microbiota composition.

References	Experimental design	Main results concerning glycaemic control	Main results concerning microbiota
Sung et al. (30)	Animals: non-germ free C57Bl/6J mice Age and sex: 8-week-old; male Diets: high-fat high-sucrose diet (45% of energy from fat and 17% from sucrose) Faecal transplantation: from mice fed a standard diet or from mice fed the high-fat high-sucrose diet supplemented with 0.4% of resveratrol Experimental period length: 8 weeks (last 2 weeks of transplantation)	Transplantation from mice fed resveratrol: Improvement in glucose clearance	↑ *Parabacteroides* ↓ *Proteobacteria* ↓ *Moryella* ↓ *Akkermansia*
Kim et al. (37)	Animals: C57Bl/6J mice Age and sex: 8-week-old; male Diets: high-fat high-sucrose diet (45% of energy from fat and 17% from sucrose) Faecal transplantation: from mice fed a standard diet or from mice fed the high-fat high-sucrose diet supplemented with 0.4% of resveratrol Experimental period length: 8 weeks (last 2 weeks of transplantation)	Transplantation from mice fed resveratrol: ↓ Fasting serum glucose levels No effect on insulin levels.	Not analysed
Wang et al. (31)	Animals: C57BL/6J mice Age and sex: 6-week-old; male Diets: high-fat diet (60% of energy from fat) Faecal transplantation: from mice fed a standard diet supplemented or not with resveratrol or a high-fat high-sucrose diet supplemented with resveratrol (300 mg/kg BW/d) Experimental period length: 18 weeks (last 16 weeks of transplantation)	Transplantation from mice fed resveratrol and the high-fat diet: ↓ Fasting blood insulin No effect on fasting glucose levels Transplantation from mice fed resveratrol and standard diet: ↓ Fasting blood glucose ↓ Fasting blood glucose insulin Improved glucose and insulin response	↑*Allobaculum* ↑ *Bacteroides* ↑ *Alistipes* ↓ *Desulfovibrio*

BW, body weight.

In this line, Sung et al. (30) fed a cohort of C57BL/6N mice a high-fat high-sucrose diet (45% of energy from fat and 17% from sucrose) for 5 weeks and then, by oral gavage, they administered three fecal matter transplantations (FMTs) during another 2 weeks, collected from donor mice fed either a chow diet (without resveratrol) or a high-fat high-sucrose diet supplemented with resveratrol (0.4%) for 8 weeks. One week after the final resveratrol-FMT dose, animals showed clear improvements in glucose clearance, whereas no effects were found in the control-FMTs animals. An improvement in glucose clearance was also observed in obese mice receiving FMT from donor mice maintained on a high-fat high-sucrose diet supplemented with resveratrol. This improvement in glucose homeostasis occurred in the absence of a reduction in body weight. Moreover, the obese mice that received the FMT showed higher levels of bacteria from the genus *Parabacteroides* and lower relative abundance of the phylum *Proteobacteria* and the genera *Moryella* and *Akkermansia*, which was similar to the results obtained with oral resveratrol administration.

In the study reported by Kim et al. (37), the authors fed C57BL/6N mice a high-fat high-sucrose diet (45% of energy from fat and 17% from sucrose) for 5 weeks. These mice were assigned either to a group receiving fecal microbiome fecal matter transplantations from donor mice fed a chow diet (FMTs) or a group receiving fecal microbiome fecal matter transplantations from donor mice fed a high-fat high-sucrose diet supplemented with 4 g of resveratrol/kg diet (Resv-FMT) by oral gavage, on days 1, 3, and 5 after an overnight fast. Mice receiving Resv-FMTs showed a marked decrease in fasting serum glucose levels, but not in insulin level, in the absence of differences in body weight. Together, these data suggest that Resv-FMTs improved glucose homeostasis by improving glucose uptake in peripheral tissues through increased sensitivity to insulin and that this effect was not secondary to weight loss. The authors tested the influence of Resv-FMTs transplant on colon inflammatory parameters in order to propose a mechanism underlying its beneficial effect on insulin sensitivity. Resv-FMT recipients showed significantly lower levels of TNF-α, CXCL1/KC (murine IL-8 homologue), and IL-1β, demonstrating reduced colon inflammation. In addition, the values obtained in a glucose tolerance test were positively correlated with the levels of TNF-α. Furthermore, the authors analyzed 23 short-chain fatty acids (SCFA) in the fecal matter of donors. This analysis revealed that the concentration of 4-hydroxyphenylacetate was significantly higher in the feces of resveratrol-fed donor mice than in the feces of the chow-fed donors. In addition, the levels of formate, isobutyrate, and isovalerate were significantly lower in the feces of resveratrol-fed donor mice. Thus, the presence or lack of these SCFAs may potentially be associated with the observed physiological benefits in obese Resv-FMT recipient mice. In this study the authors did not analyzed microbiota composition.

In a recent study, Wang et al. (31) used the feces obtained from mice treated with resveratrol at a dose of 300 mg/kg body weight/d (experiment described in the previous section of this review) to make a fecal matter transplantation to mice fed a high-fat diet. The fasting blood glucose levels were significantly lower after the transplantation from mice fed a standard diet supplemented with resveratrol. Insulin concentration was significantly lower after the transplantation from mice fed a standard diet (supplemented or not with resveratrol) and the high-fat diet supplemented with resveratrol. Moreover, glucose and insulin responses improved after the transplantation from mice receiving the standard diet (supplemented or not). Additionally, the authors confirmed a positive effect on the intestinal barrier function after microbial transplantation. When the authors analyzed microbiota composition they observed that after transplantation, the microbiota of the recipient mice resembled, to a certain degree, that of their corresponding donor groups. Thus, at the genus level, fecal-treatment increased *Allobaculum*, *Bacteroides* and *Alistipes* abundances and decreased those of *Desulfovibrio* in mice.

## Discussion

Resveratrol shows a low bioavailability, due to its low solubility in the intestine and its extensive phase II metabolism in the intestine and liver. For that reason, different strategies to increase its bioavailability have been studied. Solubility can be increased by using micronized resveratrol. In order to prevent resveratrol metabolism, this compound can be administered along with other molecules that inhibit glucouronidation and/or sulphation, such as piperine or quercetine. Another approach is to mask the prime targets of metabolism, that is the hydrophilic hydroxyl groups of resveratrol. Finally, another possibility is to use resveratrol nanoformulations (38). But, in spite of its poor biavailability, a large number of studies have shown the beneficial effects of resveratrol. One of the possible reasons is that its metabolites have biological activity, and another reason may be that resveratrol exerts its effects, in part, through the modulation of the intestinal microbiota.

In this context, and focusing the review on the beneficial effects of resveratrol on type 2 diabetes, the number of studies analyzing the effects of resveratrol on glucose homeostasis or microbiota composition are numerous. However, only a reduced number of them have addressed both issues in the same cohort of animals or humans, in order to know the potential involvement of microbiota in the anti-diabetic effects of this phenolic compound. All the studies included in this review, with the exception of that reported by Kim et al. (37) showed beneficial effects of oral resveratrol administration on glycaemic control, which is in good accordance with the vast majority of the reported studies addressing this issue (20). In the case of Kim’s work, the lack of effects could have been due to the instability of resveratrol, which, in contact with the diet, is degraded to a large extent (39). In contrast, the studies present inconsistencies in the findings concerning the effects of resveratrol on gut microbiota changes. Indeed, the changes observed in the studies are very different.

By analyzing the experiment designs used in the studies carried out in rodents, it is evident that important differences exist among parameters that play a crucial role in the influence of polyphenols on gut microbiota. One of them is the experimental period length, which is in the range 3–16 weeks. On the other hand, the used resveratrol doses and ways of administration are also different. Regarding the dose, this ranged from 15 to 200 mg/kg body weight/day of resveratrol. Moreover, resveratrol was administered either by oral gavage or included in the diet. As explained previously in this review, the diet is an important factor affecting gut microbiota composition; thus, the experimental diet may have conditioned the effect of resveratrol in this regard. The diets used in the studies were mainly high-fat diets providing 60% of energy as fat, but other types of diets, such as high-fat diets providing 45 or 72% of energy as fat, and diets rich in fat and sucrose have also been used. In addition, no information about the fatty acid composition of the dietary fat component was included in the articles included in this review. Thus, the potential differences among studies and their influence on the observed effects cannot be considered to discuss the differences observed among all the studies.

An important aspect to be considered is that, in some of these works, the authors limited the study to the description of changes in gut microbiota composition, without establishing a relationship between changes in microbiota and the improvement in glycaemic control. In other works, the authors analyzed the correlations between changes in gut microbiota and parameters related to glucose homeostasis control, but this analysis did not allow them to know if the changes observed in gut microbiota composition were a causal factor of the improvement in glucose homeostasis. Thus, unfortunately, a deeper discussion to explain which metabolic changes could be expected, based on the changes in gut microbiota, which could serve to explain the improvement in glycaemic control, was not provided.

In order to give more insight on this topic some researchers have addressed experiments based on FMT from animals treated with resveratrol to animals showing either insulin resistance or type 2 diabetes mellitus. This approach provides a stronger scientific evidence about the potential causality of microbiota composition changes. Concerning this type of studies, in that reported by Kim et al. (37) the authors did not characterize fecal microbiota composition, and thus no information concerning the potential bacteria responsible for the improvement of glucose homeostasis in mice receiving the FMTs was provided. In contrast, they analyzed 23 SCFA in the fecal matter of donors. According to this analysis, they suggested that the increased amount of 4-hydroxyphenylacetate and the decreased amounts of formate, isobutyrate and isovalerate may be responsible of the beneficial effects of Resv-FMT. In the study reported by Sung et al. (30) an increase in the genus *Parabacteroides* and a decrease in the phylum *Proteobacteria* and the genera *Moryella* and *Akkermansia* seem to be related to the improvement of glucose homeostasis induced by resveratrol. Wang et al. (31), analyzed microbiota composition and observed that after transplantation, the microbiota of the recipient mice partially resembled that of their corresponding donor groups. In this case, the changes in microbiota compostion related to the reductions observed in serum glucose and insulin levels were an increase in *Allobaculum*, *Bacteroides* and *Alistipes* abundances and a decrease in *Desulfovibrio* abundance.

As indicated in the Introduction section, certain bacteria are increased (*Lactobacillus, Streptococcus*, *Escherichia, Veillonella*, *Collinsella, Bacteroides*, *Lachnoclostridium*, *Parasutterella* and *Bifidobacterium*) and other are decreased (*Faecalibacterium prausnitzii*, *Roseburia*, *Dialister*, *Flavonifractor, Allobaculum, Alistipes, Haemophilus* and *Akkermansia muciniphila*) in pre-diabetic and diabetic subjects (human and/or rodents). Among these bacteria, *Alistipes* (31) and *Allobaculum* (31, 33) are reverse modified by resveratrol in two of the studies included in this review.

As far as *Alistipes* is concerned, although its role on glycaemic control has not been reported so far, in a study addressed in women showing gestational diabetes, statistical and computational analyses of the metagenomic data identified four potential bacterial markers associated with gestational diabetes, being *Alistipes putredinis* among them. Thus, women showing gestational diabetes presented lower abundance of this bacterium (40). The reduced abundance of *Alistipes* in two different types of diabetes, type 2 diabetes mellitus and gestational diabetes, reinforce the idea of its involvement in diabetes onset. On the other hand, it is well known that some species of this genus, such as *Alistipes onderdonkii*, seem to play an anti-inflammatory role in some diseases (41, 42). In fact, some authors observed that *Alistipes* was negatively correlated to TNF-α and lipopolysaccharide in type 2 diabetic rats, suggesting that it may suppress diabetic inflammation (43). Regarding the genus *Allobaculum*, it has been reported that calorie restriction treatment, a dietary intervention used to reduce body weight and improve insulin resistance and glycaemic control, increases its abundance. Other treatments that also improve glycaemic control, such as berberine and metformin administration (44), have also been reported to increase the abundance of this genus. Taken together, these data and those gathered in the present review, suggest that treatments able to ameliorate glycaemic control could act, at least in part, by increasing *Allobaculum* abundance.

There are two additional genera, *Desulfovibrio* and *Blautia* that decreased and increased, respectively, in some of the studies included in the present review (31, 35). *Desulfovibrio*, which is a genus of the phylum *Proteobacteria*, is known for its sulfate-reducing capacity, thereby producing toxic hydrogen sulfide, which can permeate the gut mucus barrier and increase inflammation levels (45). Taking that into account, and considering that a low-grade inflammation is associated with type 2 diabetes, the involvement of *Desulfovibrio* in the anti-diabetic effect of resveratrol could be proposed. Moreover, mice treated with liraglutide, an injectable GLP-1 receptor agonist used in the treatment of type 2 diabetes, has been shown to induce a recruitment of the genus *Desulfovibrio* (46). These findings support the potential involvement of *Desulfovibrio* in glucose homeostasis through the production of H_2_S.

Finally, it has been reported that the treatment with metformin in rodents leads to an improvement in glycaemic control, accompanied by an increase in the intestinal abundance of the genus *Blautia* in rats (44). This result has been confirmed in type 2 diabetic patients treated with this anti-diabetic drug (47). Moreover, the administration of a quality controlled herbal formula composed of eight herbs, namely, *Rhizoma Anemarrhenae, Momordica charantia, Coptis chinensis*, *Salvia miltiorrhiza*, red yeast rice, *Aloe vera, Schisandra chinensis* and dried ginger (Jiangyin Tianjiang Pharmaceutical Co., Ltd.), which induced significant amelioration in fasting glucose, HbA1c and HOMA in diabetic patients, also increased gut *Blautia* abundance (47). These results suggest that *Blautia* might be a target for the management of diabetes. In addition, it has been reported that *Blautia* is a common acetic acid producer in the intestine, which may inhibit insulin signaling in adipocytes by activating the G protein-coupled receptors GPR41 and GPR43, thus promoting the metabolism of unbound lipids and glucose in other tissues, and consequently alleviating obesity-related diseases such as type 2 diabetes. A cross-sectional study showed that *Blautia*, especially *Blautia luti* and *Blautia wexlerae*, probably help to reduce the inflammation associated with obesity-related complications. The beneficial effects of *Blautia wexlerae* are correlated with the production of S-adenosylmethionine, acetylcholine and l-ornithine and carbohydrate metabolism, resulting in the accumulation of amylopectin and production of succinate, lactate, and acetate. Consequently, *Blautia wexlerae*, modifies the gut environment, including the bacterial and SCFA composition of the gut microbiota (48).

## Concluding remarks and perspectives

In view of the results presented in the studies included in the present review, it can be proposed that four genera, *Alistipes, Allobaculum, Desulfovibrio* and *Blautia* could be involved in the positive effects of resveratrol on glycameic control. Nevertheless, it is important to emphasize that this is mainly based on studies addressed in rodents. Moreover, some limitations are observed in this field of research. First of all, the number of studies analyzing both the effects of resveratrol on glucose homeostasis and microbiota composition in the same cohort of animals, in order to know the potential involvement of microbiota in the anti-diabetic effects of this phenolic compound, are very scarce and practically inexistent in the case of humans. Moreover, the studies present inconsistencies in the findings concerning the effects of resveratrol on gut microbiota changes. In addition, the experimental designs used do not allow the researchers to establish a causal relationship between the changes in microbiota and the anti-diabetic effect, in the vast majority of the studies. Finally, the knowledge about the role of each type of bacteria on glycaemic control is not sufficient so far.

Consequently, there is a clear need for further investigation to define the actual involvement of microbiota in the anti-diabetic effect of resveratrol. The first step should be to well establish the role of different types of bacteria in glucose metabolism and other metabolic processes related to insulin resistance and type 2 diabetes mellitus such as inflammation. Moreover, a more accurate characterization of the alterations in gut microbiota composition accompanying type 2 diabetes mellitus should be addressed. In addition, large scale randomized clinical trials devoted to analyzing the effects of resveratrol on glucose homeostasis and gut microbiota in very well defined cohorts, in order to limit inconsistencies among different studies, are required.

## Author contributions

MP and IM-L: conceptualization. MP: supervision and funding acquisition. All authors: writing – original draft preparation, writing – reviewing and editing, and have read and agreed to the published version of the manuscript.
